# Biomechanical Cues Direct Valvulogenesis

**DOI:** 10.3390/jcdd7020018

**Published:** 2020-05-19

**Authors:** Neha Ahuja, Paige Ostwald, David Bark, Deborah Garrity

**Affiliations:** 1 Department of Biology, Cell and Molecular Biology Program, Colorado State University, Fort Collins, CO 80523, USA; neha.ahuja@colostate.edu (N.A.); paige.ostwald@rams.colostate.edu (P.O.); 2 Department of Mechanical Engineering, Colorado State University, Fort Collins, CO 80523, USA; david.bark@colostate.edu

**Keywords:** biomechanics, cardiac valve development, mechanotransduction, bmp signaling

## Abstract

The vertebrate embryonic heart initially forms with two chambers, a ventricle and an atrium, separated by the atrioventricular junction. Localized genetic and biomechanical information guides the development of valves, which function to ensure unidirectional blood flow. If the valve development process goes awry, pathology associated with congenital valve defects can ensue. Congenital valve defects (CVD) are estimated to affect 1–2% of the population and can often require a lifetime of treatment. Despite significant clinical interest, molecular genetic mechanisms that direct valve development remain incompletely elucidated. Cells in the developing valve must contend with a dynamic hemodynamic environment. A growing body of research supports the idea that cells in the valve are highly sensitive to biomechanical forces, which cue changes in gene expression required for normal development or for maintenance of the adult valve. This review will focus on mechanotransductive pathways involved in valve development across model species. We highlight current knowledge regarding how cells sense physical forces associated with blood flow and pressure in the forming heart, and summarize how these changes are transduced into genetic and developmental responses. Lastly, we provide perspectives on how altered biomechanical cues may lead to CVD pathogenesis.

## 1. Structure and Function of Mature Valves

Over the lifetime of an individual, valve leaflets must withstand extraordinary physical demands. Heart valves open and close as many as 40 million times a year and 3 billion times over an average lifetime. Unobstructed forward blood flow in the heart demands that leaflets or cusps maintain substantial mobility, pliability, and structural integrity. For valves to prevent backflow, the cusps must stretch and mold to fully cover the orifice, and they must resist back pressure present in the downstream anatomy of the heart. All four cardiac valves display a similar three-layered architecture, which contributes to their incredible strength, durability, and flexibility [1,2]. The mammalian vertebrate heart has four sets of valves: the aortic and pulmonary semilunar valves separate the ventricles from circulation, whereas the mitral and tricuspid valves separate the atria from the ventricles. Each of these valve leaflets has a unique physiology and structure that allows them to meet their local hemodynamic and locational challenge [3]. Valve endothelial cells (VECs) line the entire outer surface of the valve leaflet, and serve to limit inflammatory cell infiltration and lipid accumulation. Inside the valve, valve interstitial cells (VICs) are arranged in three layers, termed the fibrosa layer, the spongiosa layer, and the ventricularis layer. Cells in each layer produce a specialized extracellular matrix (ECM).

Within the fibrosa layer, densely packed collagen fibers are arranged in a circumferential orientation. Collagen fibers are primarily responsible for the load-bearing properties of heart valves. Fibrillar collagen is notable for its high tensile strength when taut, due to its intertwined triple-helix structure, but it cannot be compressed. Therefore, during systole, as the valve opens, collagen fibers adopt a crimped, corrugated, and less aligned conformation. During diastole, as the valve closes and leaflets span the orifice, collagen fibers unfold and adopt a parallel orientation. The cyclical nature of the heartbeat requires that collagen fibers be extremely sensitive to instantaneous mechanical stretch.

The spongiosa layer is organized as a porous gel matrix composed of proteoglycans interspersed with randomly oriented collagen and fine layers of elastic tissue. Proteoglycans are composed of a protein core covalently linked to smaller linear sugar polymers termed glycosaminoglycans. Due to the dense negative charges on the glycosaminoglycan side chains, proteoglycans attract and bind water abundantly. The hydrated matrix cushions compressive forces and absorbs transient stresses during valve closure. It also provides lubrication between the fibrosa and ventricularis layers and sustains the flexibility of the leaflets during constant valve motion.

Finally, the ventricularis is a thin layer enriched in radially oriented elastic fibers that provide the stretchiness necessary for the leaflets to repeatedly deform. Elastin is a long hydrophobic protein that coils to minimize exposure of its surface to water. When the valve closes during diastole, the tissue of the leaflets stretches due to backpressure; elastin fibers adopt an elongated and taut configuration, thus enhancing full valve closure. During systole, as the valve opens, elastin rebounds and the leaflet tissue bends and relaxes. The ventricularis layer is adjacent to the endothelial surface directly exposed to incoming blood flow, whereas the fibrosa layer, on the opposite side, is near the surface commonly exposed to turbulent flow just beyond the valve. Over the lifetime of the organism, the durability of the valve requires constant monitoring of the valvular extra-cellular matrix (ECM). The optimal amount, quality, and arrangement of collagen, elastin, and glycosaminoglycans contribute to the ability of cells in the valve tissue to sense their mechanical environment, and transduce that information intracellularly to mediate molecular changes. Active function of the valve requires synthesis, remodeling, and repair of ECM components, particularly collagen, elastin, and glycosaminoglycans.

In the United States, valve disease has an overall prevalence of approximately 2.5% and imposes a health care burden of approximately 1 billion dollars per year. Treatment is often limited to surgical interventions [4]. Both congenital valve disease and acquired valve disease result in ECM disorganization and VIC activation, leading to fibrotic valve leaflets that fail to ensure unidirectional blood flow, or leaflets that do not fully open (e.g., aortic stenosis), leading to disturbed flow and extra load on the ventricle. Over time, ineffectively functioning valves can lead to decreased cardiac output and cardiac failure. While the majority of valve diseases onset during adulthood, the origin of these diseases is often related to development—either through congenital malformations that grow increasingly severe with time or through the activation of normally quiescent fetal gene programs [5,6]. For example, Bicuspid Aortic Valve disease (BAV) is a common congenital malformation estimated to affect approximately 0.5–2% of people worldwide. The aortic valve usually develops with three leaflets; patients with BAV develop aortic valves with two unequal-sized leaflets. BAV is usually asymptomatic during childhood, but over time progresses to more serious conditions such as aortic valve calcification [7]. A greater understanding of the mechanisms that control valve development may inform our view of valve etiology.

## 2. Valve Development

Heart valve defects occur in 20–30% of congenital heart defects, and the incidence of valve defects has been estimated as high as 5% of live births [8]. As one example, BAV, the most common etiology to require an adult heart valve replacement, is caused by defects in the development of the aortic valve. Despite their physiological differences, the initial development of the four sets of valves is quite similar. The following section will describe the developmental processes that give rise to valve leaflets.

### 2.1. Linear Heart Tube Development

Following gastrulation, mesenchymal cells from the mesoderm migrate laterally to form separate but bilateral regions, termed the primary heart fields. Even before the heart tube forms, atrial and ventricular precursor cells already express distinct genetic programs and reside in distinct areas. Mesodermal cells from the primary heart field then migrate to the midline and coalesce into a structure termed the cardiac crescent in humans, mice, and chicks, and the cardiac cone in zebrafish. The cardiac crescent or cone eventually remodels to give rise to the linear heart tube [9], consisting of two cell layers: an outer myocardial cell layer and an inner endocardial cell layer separated by an acellular ECM termed the cardiac jelly. Valve development begins soon after linear heart tube formation.

The earliest stages of heart formation involve the specification of the cardiogenic mesoderm within the splanchnic mesoderm. This is accomplished through signals from overlying ectodermal cells as well as from a transitory cell signaling center that organizes gastrulation (termed Henson’s node in chickens and mammals, the Spemann Organizer in amphibians, and Kupffer’s vesicle in zebrafish). The anterior endodermal cells secrete paracrine factors such as Bone Morphogenetic Protein 2 (BMP2) and Fibroblast Growth Factors, which act as positive signals for cardiogenic mesoderm induction. Inhibitory signals such as Chordin, Noggin, and Wnt3a serve to restrict cardiac induction to a subset of the splanchnic mesoderm. This combination of signaling factors serves to induce expression of cardiogenic transcription factors such as T-box transcription factors, Tal1, and GATA. These regions of specified cardiogenic mesoderm are termed the bilateral primary heart fields [9].

Cardiac progenitor cells must translocate from the bilateral heart fields to the body midline and reassemble to form the cardiac crescent (or cone). The migratory process is facilitated by endocardial cells and their associated extracellular matrix. Current research suggests that endocardial cells originate from a specific population of mesodermal cells within the primary heart field [10]. Migration from the heart field to midline and subsequent fusion is regulated by a combination of paracrine signaling and extracellular matrix proteins. For example, knockdown of the paracrine factor *vegfa* or its receptor leads to failure of endocardial tube fusion in zebrafish [11]. Additionally, it is thought that expression of the myocardial gene *Tmem2* creates an extracellular matrix environment that facilitates endocardial cell migration [12].

After endocardial cells assemble into a midline endocardial tube, myocardial cells are recruited around the endocardial cells to form a two-layered heart tube. In contrast to endocardial cells—which migrate as individual cells—evidence from zebrafish embryos indicates that myocardial cells migrate as a sheet [13]. This process is regulated by ECM components, such as fibronectin, as well as by communication from the adjacent endodermal cells. For example, disruption of platelet-derived growth factor (PDGF) through mutation of its receptor, *pdgfra*, prevented myocardial migration towards the midline in zebrafish embryos. The PDGF ligand is expressed by endodermal cells at the midline of the embryo and acts as an instructive cue to direct myocardial cell migration [14]. Fibronectin is a multi-domain glycoprotein that gradually assembles into an insoluble fibrillar matrix by binding other fibronectin proteins, and ECM components including collagens, proteoglycans, and integrins. Knockdown of fibronectin prevents myocardial cell migration in zebrafish embryos and mouse embryos [13,15]. In zebrafish *cloche* mutants, which lack the endocardial cells that produce fibronectin, the myocardial cells still initiate migration, but are unable to assemble into a cardiac cone at the midline [16]. This observation suggests that signals from endocardial cells facilitate the directional migration of myocardial cells, though the molecular mechanisms behind the process are unclear. The correct completion of this process results in the formation a two-layered heart tube.

### 2.2. Endocardial Cushion Formation

**Figure 1:**
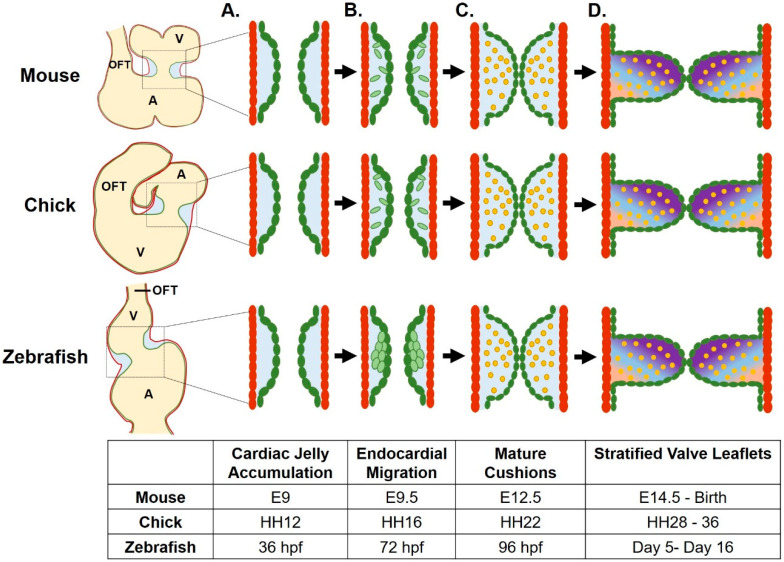
Atrioventricular valve development across species. (**A**) Valve development begins as cardiac jelly accumulates between the myocardial (red) and endocardial cell layers. (**B**) Endocardial cushions (ECs, in green) are created as endocardial cells invade the cardiac jelly following epithelial to mesenchymal transition (mouse, chick) or via a local invagination of the endocardium (zebrafish). (**C**) ECs mature as the mesenchymal cells differentiate into valve interstitial cells (VICs, in yellow) as the cushions begin to function to occlude retrograde flow. (**D**) Valve leaflets form from remodeled ECs, as VICs proliferate, stratify, and differentially modify the extracellular matrix (ECM). Gradient represents three layers of stratified ECM. A, atrium; V, ventricle; OFT, outflow tract. Figure created using BioRender.

After formation of the linear heart tube, the heart initiates cardiac looping and ballooning. During these processes, the linear heart tube grows in cell number and begins to fold into an S-shape that evolves into a two-chambered heart. Concomitantly, the endocardial cushions, which are thickened mounds of specialized ECM in association with endocardial cells, accumulate at the atrioventricular junction and at the outflow tract. The endocardial cushions, in association with adjacent endocardial cells, give rise to the valve leaflets at these two locations [8]. The following sections describes current knowledge regarding development of the endocardial cushions (Figure 1).

The first step in the formation of the endocardial cushions is the accumulation of the cardiac jelly at the atrioventricular canal. The cardiac jelly is a layer of ECM located between the endocardial and myocardial layers. Initially, the cushions consist primarily of a hydrated network of proteoglycans (such as versican) and glycosaminoglycans (such as hyaluronan). Over time, structural proteins such as collagen and fibronectin are deposited as well. Disruptions of ECM proteins results in defective endocardial cushion formation. For example, knockdown of *hyaluronan synthase 2*—the enzyme that synthesizes hyaluronan—results in fewer valve-forming cells at the atrioventricular junction in zebrafish embryos [17]. In addition, morpholino knockdown of *fibronectin 1b* results in defective endocardial cell clustering at the atrioventricular junction [18]. Similarly, murine embryos with mutations in fibronectin showed defective endocardial cushion formation and lacked differentiated cushion cells [19]. While the exact function of atrioventricular junction accumulation of cardiac jelly in valve development is not known, current models suggest that the ECM creates a permissive environment that allows further progression of endocardial cushion development [20].

As the ECM at the atrioventricular junction differentiates, the adjacent endocardial cells proliferate and migrate into the cardiac jelly to populate the endocardial cushions. In mammalian hearts, this event is accomplished through an endothelial-to-mesenchymal transition (EMT), a complex event involving over 100 genes. Activated endothelial cells reduce their endothelial markers, gain mesenchymal markers such as *α-smooth muscle actin* (*α-SMA*), and release their contacts with neighboring cells. Soon, endothelial cells delaminate from the endocardial layer, acquire a mesenchymal phenotype, and invade the cardiac jelly. Once in the cardiac jelly, these cells proliferate and differentiate into VICs, which gradually stratify to give rise a functional valve leaflet. In zebrafish, leaflet formation initiates with a local invagination of the intact AV endocardial layer into the cardiac jelly [21] (Figure 1). Once folding occurs, cells then delaminate and invade the cardiac jelly [18,22]. The signaling mechanisms mediating mammalian and zebrafish endocardial cushion formation and remodeling are quite complex, and involve extensive reciprocal communication between the endocardium and the myocardium, as well as the intersection of several signaling pathways [8].

Recent data suggests that the Bone Morphogenetic Protein (BMP) pathway plays a key role in coordinating endocardial cushion development [8]. BMP ligands are paracrine factors that have extensive roles in regulating cardiac morphogenesis, reviewed in [23]. Of particular importance are BMP2 and BMP4, which are secreted by the myocardial cells and which are required for induction of EMT in the overlying endocardial cells. The first evidence supporting a role for BMP in valve formation came from in vitro experiments in which endocardial cells were cultured upon gel monolayers. Mouse endocardial cells did not undergo EMT when cultured alone; however, when cultured with exogenous Bmp4, endocardial cells abolished PECAM expression and acquired a mesenchymal phenotype [24]. Since this seminal work in 2004, a requirement for BMP in EMT has been verified in several in vivo models. The mutation of *bmp4* in mouse models resulted in hypocellular endocardial cushions [23]. Similarly, knockout of *BMP2* in mouse embryos resulted in failure of cardiac jelly expansion at the AVJ, and abrogated EMT [25]. In zebrafish, chemical inhibition of *bmp2/4* similarly resulted in failure of endocardial cell differentiation [26].

**Figure 2:**
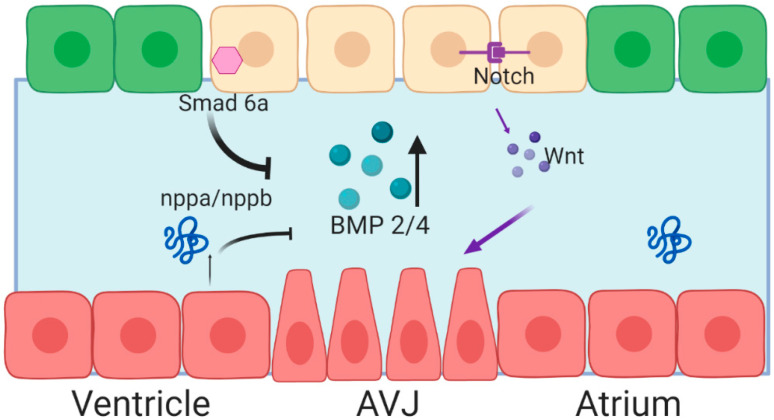
Spatiotemporal expression of Bone Morphogenetic Protein (BMP) 2/4 is achieved through activation at the AVJ, and repression through the cardiac chambers. Red cells represent myocardial cells, green cells represent endocardial cells, yellow cells represent valve-forming endocardial cells. Blue space between the two cell layers represents the cardiac jelly. Figure created with BioRender.

The requirement for Bmp4 in endocardial cushion formation has been well established; however, several open questions remain regarding the establishment of Bmp signaling in the cardiac valve. In zebrafish, the dynamic pattern of *bmp4* expression is rather curious—*bmp4* is first expressed throughout the myocardium of the entire linear heart tube. At 36 hpf in zebrafish, *bmp4* expression retracts to the atrioventricular junction myocardium [27]. Likewise, in mouse embryos, BMP2 and BMP4 are both expressed throughout the cardiac crescent, and the expression becomes restricted over time. Interestingly, their expression patterns diverge over time; by E12.5, BMP2 is expressed in the AVJ and outflow tract mesenchyme, while BMP4 is expressed exclusively at the OFT [28]. The establishment of this restricted expression pattern is dependent on both paracrine signaling and extracellular matrix proteins (Figure 2).

Expression of BMP2 and BMP4 is positively regulated by canonical Wnt signaling. In mouse embryos, Notch signaling coordinates endocardial Wnt4 expression, which in turn regulates myocardial expression of BMP2 [29]. In zebrafish embryos, repression of Wnt signaling through overexpression of *axin*, a protein involved in the degradation of β-catenin, abrogated *bmp4* expression at the AVJ [30]. In addition to positive signaling, inhibitory signaling represses *bmp4* expression in the ventricular myocardium. Knockdown of the extracellular matrix protein, Nephronectin, in zebrafish embryos results in expanded Bmp4 signaling and impaired endocardial valve cell differentiation, suggesting that Bmp signaling can be modulated by the ECM [17]. Chamber myocytes secrete Atrial neurotic peptide (Anf/Nppa), which serves to repress BMP expression. Additionally, Smad6, an inhibitory SMAD, is expressed in the valve endocardium, putatively as part of a negative feedback loop to control BMP signaling. Homozygous mice lacking Smad6 (Madh6) generated an excess of AVJ mesenchymal cells at the time of EMT and later developed hyperplastic valves [31]. Knockout of Smad6 resulted in dramatic reorganization of the ECM—specifically, *periostin* and *veriscan* expression were significantly reduced in mutant mice [32]. Together, these data highlight the complex combinatorial control that constrains the region of BMP expression and triggers further valve cell differentiation.

### 2.3. Mesenchymal Cells Populate the Endocardial Cushions

Endocardial cushions undergo substantive remodeling to give rise to the mature valve leaflet. This process is primarily characterized by alterations in the ECM. The ECM of endocardial cushions consists primarily of proteoglycans, while the valve leaflet ECM consists of three stratified layers: a collagen-rich layer termed the fibrosa, a proteoglycan-rich layer termed the spongiosa, and an elastin-rich layer termed the atrialis in the AV valves, or the ventricularis in semilunar valves [8,33]. Heart valve development is characterized by increasing complexity and organization within the ECM. As mesenchymal cells populate the endocardial cushions, they proliferate, migrate, differentiate into fibroblastic interstitial cells and remodel the ECM.

Surprisingly, EMT-derived mesenchymal cells continue to express cadherin-based adherens junctions including N-cadherin and Cadherin-11 at their surface for a brief period while colonizing the cardiac jelly. Mesenchymal cells display a collective migration and intercellular communication that is important to this process. Mice with Cadherin-11-depleted valve cells completed EMT successfully, but were unable to migrate collectively, showed defects in protrusion formation and failed to populate the cushions evenly [34]. Downstream of Cadherin-11, RhoA activity supports the migratory behavior and traction force required for morphogenesis.

How endocardial cells choose to become VICs is a topic of intense interest. Studies in zebrafish embryos indicate this cell fate decision is regulated by *Nuclear Activated T-Cell Factor (NFAT)*, as well as an EMT-promoting transcription factor, *twist1b* [35]. By 144 hpf in zebrafish embryos, cells in the ECM of the atrioventricular valve no longer express the endocardial marker *fli1a*, but actively express *hhex*, a marker for VICs. Lineage tracing experiments using the photoconvertible transgene kaede in conjunction with reporter lines for either neural crest (*sox10*), endocardium (*kdrl*), or epicardium (*tcf2i*) demonstrated that VICs predominately originate from the endocardial lineage with a small contribution from the neural crest. As early as 38 hpf, *nfat* is specifically expressed in the atrioventricular junction endocardium. Lineage tracing of photoconverted cells at 38 hpf revealed that *nfat*-expressing endocardial cells give rise to approximately 80% of the VICs present at 86 hpf. Knockout of *nfat1* in zebrafish embryos abrogated VIC formation and decreased proliferation by 72 hpf in the valve endocardium. Interestingly, *nfat1* mutants displayed a significant decrease in the expression of *twist1b*. Overexpression of a dominant negative *twist1b* allele prevented valve leaflet entirely. Taken together, this demonstrates that endocardial cells make decisions regarding VIC cell fate early on in cardiac development, and that this process requires NFAT [35].

Recent work in mice demonstrated that following EMT, mesenchymal cells activate expression of the Sox9 transcription factor in the population of cells that differentiate as VICs [36]. In embryonic stages, Sox9 is not expressed in VECs, and thus may provide a genetic handle to identify additional genes required for VIC differentiation. A critical role for Sox9 in proliferation of valve precursor cells was suggested by targeted loss of function mice, which display severely hypoblastic endocardial cushions [37]. Sox9 has later embryonic roles in ECM remodeling later in valve development, and following birth, becomes expressed in VECs as well as maturing VICs [36].

### 2.4. Neural Crest Cells Contribute to the Development of the Semilunar Valves

Neural crest cells contribute to the development of the outflow tract and consequently the development of the aortic and pulmonary valves. At E8.5 in mouse embryos, cardiac neural crest cells delaminate from the neural tube and migrate to the outflow tract, where they differentiate into smooth muscle cells that contribute to the aortic arch. By E10.5, endocardial cells lining the outflow tract undergo EMT and begin to invade the outflow endocardial cushions. Concomitantly, neighboring cardiac neural crest cells migrate in bilateral columns into the outflow cushions, where they give rise to a portion of the VICs [38]. Defective neural crest cells produced by loss-of-function mutation of the Pax6 transcription factor led to the development of dysmorphic, nonfunctional semilunar valves [39]. Similarly, laser ablation of neural crest cells in quail embryos at HH8 led to dysmorphic valves [40]. Defects in neural crest cells are strongly associated with a host of CHDs—including DiGeorge’s Syndrome, Noonan’s syndrome, and bicuspid aortic valve disease—extensively reviewed in [41]. Together, this data strongly suggests that presence of neural crest cells in the outflow tract is essential for proper semilunar valve development.

Reciprocal signaling from neural crest cells and other neighboring cell types in the heart field may aid the patterning of semilunar valves. Neural crest cells are thought to aid in the development of valve leaflet ECM as well as facilitate apoptosis of mesenchymal cells in later stages of gestation. Deletion of *Pax3*, a neural crest cell marker, resulted in abnormal hypercellular and thickened aortic valve leaflets in mouse embryos at E17.5. As neural crest cells migrate into the outflow tract, they pass by cells of the secondary heart field, presenting an opportunity for paracrine or juxtacrine signaling. Tissue-specific inhibition of *notch1* from the secondary heart field phenocopied the malformed valves and altered neural crest cell patterning of *pax3* mutants [39]. Tissue-specific inhibition was accomplished through expression of a well-characterized Notch inhibitor, DNMAML, in conjunction with an *Islet1^Cre+^* line to constrain expression specifically to the second heart field. Likewise, tissue-specific haploinsufficiency of *notch1* in endocardial cells in a Nos3^-/-^ background resulted in a similar bicuspid aortic valve phenotype, whereas deletion of *notch1* from neural crest did not produce an obvious phenotype [42]. Together, this data highlights intracellular communication via *notch1* as a key regulator of neural crest cell migration and valve leaflet formation.

The presence of neural crest cells may provide an instructional cue that aids in defining the geometry of the tricuspid valve leaflet. At E10.5 in mouse embryos, neural crest cells within the cardiac outflow cushions are distributed throughout the circumference of the cardiac jelly, and begin to aggregate. Condensation refers to the process of aggregated cells becoming aligned and attached via cell:cell contacts. By E11.5, two distinct condensed clusters are evident, termed the superior and inferior cushions. Mice bearing a dominant negative mutation of *ROCK* (a Rho Kinase) showed uneven positioning of neural crest cells in cardiac jelly, followed by disruptions in the condensation process and ultimately, abnormal aortic valve leaflet morphology. Specifically, when dominant negative *ROCK* mutation resulted in fewer or hypoplastic neural crest cell clusters, the aortic valves developed a bicuspid valve leaflet phenotype. When the same mutation resulted in multiple patchy, aberrant cell clusters, the aortic valve adopted a quadriscuspid phenotype. Together, this data strongly suggests that proper positioning of neural crest cells directs the formation of the outflow cushions, and each individual valve leaflet [43].

Several signaling pathways control the differentiation and activity of the valvular neural crest, including the *Bmp4*, *Fgf8*, and *Hippo* pathways [44,45]. Recently, *Krox20*, a transcription factor that regulates collagen, was identified as a cell-autonomous factor that regulates neural crest cell apoptosis. Fate mapping studies using Krox20^Cre^ transgenic mice demonstrated that Krox20 is specifically expressed in the subpopulation of neural crest cells that invade the arterial valve cushions. By E18.5, homozygous Krox20 mutant mice display dysmorphic, hypercellular aortic valve leaflets with disorganized ECM and a partially penetrant bicuspid valve phenotype. Interestingly, endothelial-derived VICs were present in normal numbers, but neural-crest derived VICs were increased approximately 1.8-fold in Krox20 mutants as compared to stage-matched wildtype embryos. Together, this data demonstrates that *Krox20* is a key transcription factor regulating neural crest cell behavior and valve leaflet formation [46].

### 2.5. Valve Extracellular Matrix Remodeling

After formation of the VICs, valve leaflets must remodel their existing ECM into the tri-layered adult valve leaflet structure. The mechanisms that control this process during development are not well understood; however, some insight can be gained from the study of valve leaflets in adult humans or model organisms. Throughout infancy and adolescence, hemodynamic pressure within the heart increases as the circulatory system expands to meet increased physiological demands. Valve leaflets undergo extensive growth and remodeling in order to meet these demands [47]. Tissue growth, including changes in leaflet length, area, and thickness, incurs the production of new ECM. Tissue remodeling, however, occurs via changes in ECM structure and composition. In particular, leaflets develop thicker collagen bundles and denser collagen cross-links. These changes in collagen architecture serve to increase stiffness of the leaflets with age. In adult humans with high blood pressure, similar remodeling events are mediated by altered hemodynamics and can lead to valvular disease [48]. Whether leaflets in adults respond to the same mechanisms as were active in the initial valve leaflet development remains relatively uninvestigated. Mutations in either collagen or elastin are linked to a variety of severe congenital heart defects [7]. During late gestation, apoptosis is used to sculpt the leaflets. A significant percent of the mesenchymal cells undergo apoptosis, leaving a valve leaflet that consists of a tri-layered ECM with relatively few VICs [49].

## 3. Biomechanical Inputs in Valve Development

The heart is the first organ to form and begins contractions shortly after formation of the linear heart tube. Cardiac contractions begin at 22 hpf in zebrafish, 48 hpf in chicks, embryonic day 8.5 in mice, and at approximately 22 days in humans. As development progresses, heart contractions increase in force and frequency, thereby altering hemodynamic forces. Both blood pressure and heart rate increase rapidly as differentiation proceeds in zebrafish, chicken, mouse, and humans [50]. As hemodynamic factors change both spatially and temporally, they can elicit precise transcriptional responses. The following section will review current knowledge regarding the role mechanical forces play in heart development.

### 3.1. Shear Stress

Conceptually, shear stress is the frictional force per unit area between laminar layers of fluid and is known as wall shear stress (WSS) at the surface of the endothelial layer as blood flows parallel to the heart wall. WSS creates a drag force on the endothelial layer and it regulates several specific aspects of normal valve development and blood vessel development, as well as pathological aspects such as fibrosis under conditions of heart failure. Both the direction and the magnitude of WSS play a role in triggering gene expression changes. This next section will describe the evidence from zebrafish, chicken, and mice that relate signals provided by WSS to the underlying molecular circuitry.

In vitro experiments first elucidated the role of WSS in heart morphogenesis. When exposed to laminar flow, cultured endothelial cells realigned in the direction of flow, and altered ECM production and gene expression. In particular, cells increased their expression of the transcription factor *Kruppel-like factor 2* (*KLF2*) [51,52]. The first in vivo evidence for shear stress as a factor directing valve morphogenesis came from a zebrafish model in which blood flow had been occluded in the heart by the insertion of glass beads [53]. Specifically, a glass bead placed at either the outflow or inflow tract of the developing zebrafish embryos disrupted flow at the atrioventricular junction and the rest of the heart. Under conditions of absent flows, the endocardial cushions and the atrioventricular valve failed to form [53]. Though this hemodynamic intervention is rather invasive, this work established that the presence of blood flow and associated WSS is required for normal valve development.

An open question in the field concerns the underlying molecular circuitry that regulates the WSS response. On a transcriptional level, the best studied response is the flow-responsive nature of *klf2* transcription. Under conditions of normal flow, chicks, mice, and zebrafish embryos all upregulate *klf2* transcripts in response to increased shear stress, thus triggering the activation or repression of a number of downstream signaling pathways. KLF2 is required for endocardial valve cell differentiation and endocardial cushion formation in both mice and zebrafish embryos [54,55,56].

#### 3.1.1. The KLF2 Pathway

Much of the work that elucidated the KLF2 pathway in valve development comes from zebrafish. Due to a genome-wide duplication event, zebrafish encode two paralogs of KLF2: *klf2a* and *klf2b*. The flow-responsive properties of *klf2a* expression were noted in zebrafish embryos treated to alter blood viscosity [56]. The *gata1* and *gata2* transcription factors are required for hematopoiesis. Morpholino knockdown of either of gene resulted in decreased hematocrit. While *gata1* and *gata2*-morpholino-injected embryos both displayed reduced blood viscosity, they differed in their specific patterns of blood flow and shear stress. *Gata1* morphants displayed increased oscillatory flow at the atrioventricular junction, whereas *gata2* morphants displayed reduced oscillatory flow. As these knockdowns displayed similar decreases in overall blood viscosity and shear stress magnitude but display different directional flow profiles, they provide a convenient tool to probe the role that directional shear stress plays in valve development [56].

*Gata2* morphants, with decreased retrograde flow, showed defective invagination of valve leaflets and decreased numbers of endothelial cells in the AV canal. These phenotypes could be recapitulated through knockdown of *klf2a* [56]. This data established *klf2a* transcription as responsive to oscillatory flows and required for valve development. Further work with a *klf2a* mutant showed that Klf2a function is required for cell clustering at the atrioventricular junction, as well as cell migration into the cardiac jelly. Intriguingly, a Tg[klf2a:H2B-eGFP] reporter line revealed that cells at the atrioventricular junction display a gradient of *klf2a* expression. Cells initially express high levels of *klf2a*, but as they enter the cardiac jelly *klf2a* expression becomes low. Once inside the cardiac jelly, cells acquire a putative mesenchymal phenotype and begin to proliferate [18]. Together, these data establish that oscillatory WSS regulates *klf2a* expression, which, in turn, regulates AV endocardial remodeling and cell migratory behavior.

In addition to its role in cell migration, Klf2a regulates multiple genetic pathways and morphogenetic events. Zebrafish studies demonstrate Klf2a positively regulates fibronectin, a critical extracellular matrix component [18]. In human umbilical vein endothelial cells (HUVECs), overexpression of *klf2a* produced a transcriptional profile with alterations in pathways involved in cell migration, inflammation, and stress fiber formation [57]. In mouse embryos, KLF2 expression induced WNT9b, which is required for EMT and endocardial cushion formation [55]. More work needs to be done to identify Klf2 targets in order to fully understand how Klf2 coordinates multiple morphogenetic events.

While data support the regulation of *klf2a* transcription by oscillatory WSS, how cells are able to sense oscillatory shear stress and transduce a mechanical signal into a genetic response remains an area of intense interest. One mechanism enabling sensation of oscillatory flow employs two mechanosensitive calcium channels, Trpv4 and Trpp2 [58]. These channels are both expressed in endocardial membranes facing the cardiac lumen. Mutation of either channel in zebrafish resulted in decreased *klf2a* expression at the AVC and altered valve morphology. It is proposed that, as these channels become activated by oscillatory flow, they trigger an influx of calcium into endocardial cells within the AVC, which in turn regulates *klf2a* expression [58].

In addition to mechanical regulation, KLF2 expression is also regulated by molecular feedback loops. One such feedback loop elucidated in zebrafish valvulogenesis involves the proteins *Krit1* (also known as CCM1) and *heart of glass* (HEG1). The Krit1 and Heg1 proteins bind one another and are active in the formation and stabilization of endothelial cell junctions. *Heg1* expression is regulated by blood flow and *klf2a*, and consequently is expressed at the atrioventricular junction. Heg1 stabilizes expression of its binding partner, Krit1, which is expressed in a blood flow-independent manner in the endocardium. Loss of Krit1 leads to aberrant *klf2a* expression throughout the endocardium and defects in chamber and valve formation. Together, this data suggests a model wherein *klf2a* is spatially regulated by a negative feedback loop mediated by the Krit1-Heg1 proteins [59].

Overall, the data points to a model wherein oscillatory WSS induces expression of KLF2, which subsequently mediates valve development by promoting endothelial cell reorganization and migration and by regulating extracellular matrix proteins. To achieve precise spatiotemporal regulation of KLF2 expression, both blood flow-dependent and -independent mechanisms are used in the chamber endocardium, leading to precise control of endocardial cushion and valve leaflet formation.

#### 3.1.2. The Role of Cilia in Sensing Shear Stress

Primary cilia are solitary, immotile protrusive organelles that function as mechanosensors. The stiffness of this antenna-like organelle contributes to its high sensitivity to WSS and facilitates its ability to function as a flow sensor in a variety of tissues, including Henson’s node, kidney epithelium, bile duct epithelium, and vascular endothelium [60]. As blood or fluid flows over vascular endothelium, primary cilia projecting from the luminal side of the cells deform/bend, eliciting the rapid opening of polycystin-2 calcium-permeable cation channel complexes and a subsequent influx of calcium ions. In this way, cells translate the mechanical stimulation of primary cilia into an intracellular calcium transient. Calcium influx regulates a variety of downstream signaling pathways leading to functional responses. For example, calcium levels indirectly activate eNOS, the enzyme responsible for production of nitric oxide (NO). NO exerts vasodilatory effects by promoting smooth muscle relaxation. Several studies now support cilia dysfunction as a causative component of several cardiac and vascular diseases [61].

While the role of primary cilia in vasculature development has been well-characterized, less is known about how cilia may function in endocardial or valve development. In murine models, mutations in the cilia structural gene *ift88* result in cardiac dysfunction and hypoplastic endothelial cushions, suggesting that WSS may act on cilia in the endocardium to facilitate endocardial cushion development [62,63]. In zebrafish embryos, cilia are required for activation of Notch in the endocardium [64]. The exocyst is a molecular trafficking complex required for ciliogenesis. Disruption of EXOC5, a linker protein required for exocyst integrity, produced cardiac phenotypes reminiscent of biaortic valve disease in both zebrafish and mice embryos [65]. Together, this data highlights the fact that cilia have an evolutionarily conserved role in valve development.

The integrity of cilium itself is unable to withstand high levels of shear stress. Exposure to high WSS triggers disassembly of its internal microtubule structure, the axoneme. Accordingly, cilia are normally internalized from the cell surface in areas of high shear stress such as the atrioventricular endocardium in both chick and zebrafish embryos [10]. When cultured in vitro and in the presence of laminar flow, ciliated endothelial cells do not readily undergo EMT. In contrast, genetically modified endothelial cells that lack cilia acquire a mesenchymal phenotype when exposed to laminar flow. This data strongly suggests that absence of cilia—caused by patterns of high shear stress—is a prerequisite for the transition to a mesenchymal status observed in endocardial cushion maturation [66].

#### 3.1.3. MicroRNAs in the Shear Stress Response

The role of microRNAs in regulating the shear stress response pathway has well been described in the vasculature (extensively reviewed in [67,68]). In adult cardiac valve pathology, microRNAs are emerging as novel regulators of the shear stress response. Less is known about whether or how microRNAs function to convey information about shear stress during valve development, though it is possible that the same underlying molecular circuitry is involved.

MicroRNAs are implicated in the progression of adult valve disease. For example, in calcified aortic valve disease (CAVD), the aortic valve (located between the aorta and the left ventricle) becomes gradually thicker. Over time, the aortic valve accumulates calcium deposits and obstruction of flow can occur. The aortic valve experiences low and oscillatory WSS shear on the aortic side (the fibrosa), and high WSS on ventricular side (the ventricularis). Aortic valve calcification occurs most often on the fibrosa side while the ventricularis remains relatively unaffected, making CAVD a side-specific disease. Remarkably, several microRNAs demonstrate enriched expression in the fibrosa relative to the ventricularis in healthy tissues. Ex vivo experiments demonstrate that the one-sided differential expression of microRNAs is indeed linked to oscillatory shear stress, normally larger on the fibrosa relative to the ventricularis. An attractive hypothesis is that disrupted expression of side-specific microRNAs might mediate CAVD pathology. Silencing of *miR-214* by anti-miR-214 generated increased expression of *TGFα1* in the fibrosa of porcine aortic valve explants, but only when exposed to conditions of oscillatory flow [69]. Thus, *miR-214* exhibits a shear-dependent functional role in the AV. Pharmaceutical inhibition of another microRNA, *miR-34a*, resulted in ameliorated aortic valve calcification in a murine model. *MiR-34a* is produced in human adult patients with CAVD [70].

In bicuspid aortic valve (BAV) disease, the malformed geometry of the adult aortic valve results in altered hemodynamics including abnormal patterns of shear stress and greater potential for turbulent flow. Bioinformatic analysis indicated that a number of microRNAs exhibit differential expression in the cusps of the bicuspid aortic valves, or in circulating serum, as a result of BAV disease [71]. These cohorts of microRNAs may serve as clinical biomarkers specific for BAV, and analysis of their putative targets can provide insight into the biological processes they influence. Together, these data establish that shear-stress-mediated modulation of circulating microRNAs has important impacts on molecular circuits pertinent to aortic valve disease.

The role of shear stress-mediated microRNAs is increasingly evident in adult valve pathology, but their role during normal development remains understudied. A study by Banjo and colleagues (2013) demonstrates how a flow-dependent microRNA contributes to normal valvulogenesis. In zebrafish, *miR-21* acts as a shear-responsive regulator of valve development. Arresting the heartbeat for as little as 12 h in embryogenesis was sufficient to eliminate *miR-21* expression in AVC endocardial cells, suggesting that this miR is blood-flow dependent. Levels of *pre-miR-21-1*, one of the two primary transcripts of zebrafish *miR-21*, was likewise dependent on the presence of a heartbeat, suggesting that regulation of *miR-21* biogenesis occurs at the level of transcription. Following the loss of *miR-21* expression due to heartbeat cessation, as little as 1 h of reactivated heartbeat was sufficient to restore *pre-miR-21* to normal levels. Thus, detection of flow information and the transcriptional response can be extremely rapid. Epinephrine-induced vasoconstriction doubled the shear stress and nearly doubled *pri-miR-21* expression in whole zebrafish bodies. Conversely, knockdown of *miR-21* through morpholinos produced a valveless phenotype by 72 hpf. Morphant hearts displayed few valve-specified ALCAM-expressing cells and only a single thin layer of endocardial cells in the AV canal. *miR-21* negatively regulates *sprouty2*, an inhibitor of the RTK/Ras/ERK pathway, and positively regulates several genes active in cell proliferation. Thus, *miR-21* is an important mediator of the shear-response circuitry required for valve development [72].

Several open questions remain regarding the role of microRNAs in valve development. First, are the microRNAs that are differentially expressed in a shear-specific manner in adult valve pathology also expressed during development? If so, their tightly regulated expression may constitute one mechanism that produces physiological differences between valve leaflets. Second, how is microRNA activation by shear stress achieved? Is their expression mediated by mechanosensitive ion channels, cilia, or by deformations in the glycocalyx? These questions must be addressed to fully define the molecular circuitry underlying the shear stress/miRNA axis in valve development.

### 3.2. Pressure as a Hemodynamic Cue in Valve Development

**Figure 3:**
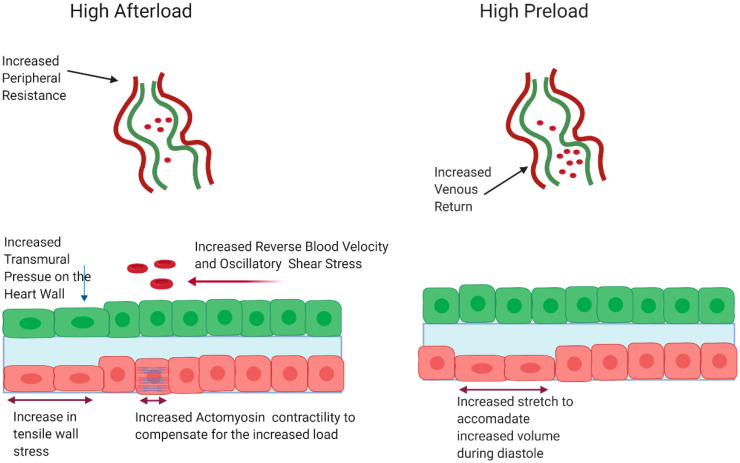
High afterload induces oscillatory shear stress, compressive pressure, and increased contractility resulting in increased tensile wall stress. High preload leads increased stretch to accommodate higher blood volumes during diastole. Figure created with BioRender.

While the biomechanical signals from WSS are becoming increasingly well characterized, less is known about how pressure impacts heart valve development. Pressure throughout the heart is determined by the loading state of the heart. Afterload refers to the pressure the ventricle must overcome to pump blood throughout the circulatory system. For a given cardiac output, the pressure is proportional to the flow rate times the resistance. The higher the afterload, the higher the transmural pressure throughout the heart [73]. In contrast, preload is defined as the degree of ventricular stretch at the end of ventricular filling. As preload increases, stroke volume and cardiac output increase concomitantly. Preload is commonly estimated by measuring left atrial pressure at the end of diastole. Preload can be thought of as passive tension placed on the ventricle while it is filling, whereas afterload is the wall stress the ventricle experiences as it is contracting (Figure 3).

As the heart develops, both preload and afterload increase to meet the demands of the embryos [74]. We and others characterized the pumping dynamics of zebrafish embryos as they progress from a linear heart tube stage (30 hpf) through looping and chamber ballooning (48 hpf). At 30 hpf, the heart tube contracts in peristaltic-like fashion, with relatively low blood velocity. As development progresses, the heart transitions to independent contraction of the atrial and ventricular chambers, deploying a displacement pumping mechanism [75,76]. This transition in pumping mechanism is recapitulated in vertebrate model organisms and serves to increase blood velocity. As an organism grows, a larger cardiac output is required and peripheral vascular resistance increases. As a result, ventricular pressure must also increase to maintain perfusion through the vascular beds [77]. Indeed, hearts in chick embryos exhibit a dramatic increase in hemodynamic pressure once valve leaflets form [78].

The increased pressure on the valve as development proceeds suggests the hypothesis that pressure regulates developmental processes that give rise to the valve leaflet. To understand how changes in pressure results in both pathological and developmental responses, it is useful to look at how pressure elicits changes on the cellular level. Increases in afterload result in transmural pressure on the cardiac wall and valvular structures. This can be mechanically transduced through deformations of the glycocalyx, β-integrin signaling, or via activation of mechano-gated ion channels [48,79,80]. In addition to the increase in transmural pressure, cardiac myocytes must increase contractility to compensate for the increased afterload, overall increasing tensile stress in the wall (not necessarily stretch, which is otherwise seen for preload). Initially, the capacity to increase contractility is accomplished through alterations in the sarcomere shortening fraction [81]. This alteration in sarcomere mechanics can be transduced to genetic responses through a variety of stretch sensitive and titin-mediated mechanisms [82]. Over time, compensation may be achieved through the initiation of the hypertrophic gene program and remodeling of the myofibril architecture. Increases in preload are accomplished through in increases in venous return. This causes an increase in tension on the atrial myocytes as they stretch to compensate for increased volume. Increases in tension can be transduced to changes in gene expression through focal adhesion kinase and other adhesion proteins [83]. The next section will review current knowledge regarding the role pressure plays in regulating valve development and pathology and will highlight mechanotransducive circuits involved in valve development.

#### 3.2.1. Pressure in Adult Pathology

High afterload exerts deleterious effects in adults that lead to cardiac hypertrophy and valve pathology [84,85,86]. Recently, two epidemiological studies involving millions of adult subjects definitively established an association between elevated blood pressure (hypertension) and regurgitant mitral valve disease, aortic stenosis (AS), and aortic regurgitation (AR) [87,88]. Each 10 mmHg increase in blood pressure was associated with a 41% higher risk of AS and a 38% higher risk in AR. As hypertension increases the pressure the ventricle must pump against, it can be thought of as a condition of high afterload. While these studies did not investigate the mechanisms that underlie the association, the authors postulate that high blood pressure exerts mechanical stress. The stress of these interactions then facilitates endothelial cell damage and altered extracellular matrix. Gradually, further structural changes impede normal function of the valve. In the case of AS and AR, the aorta becomes less distensible and the aortic valve stiffens [87,88].

**Figure 4:**
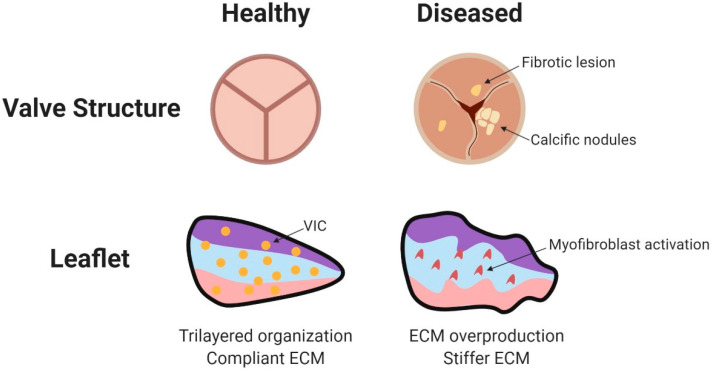
Disease pathology of adult valves. Diseased valves may exhibit calcification and fibrotic lesions. Accumulating pathology leads to leaflet calcification, valvular insufficiency, valvular stenosis, and increased cyclic wall strain. Within the valve leaflets, VICs transform to activated myofibroblasts. The increased production of collagen, elastin, GAGs, and matrix metalloproteases changes the architecture of the leaflet. Major mechanosensors in valvular endothelial cells include ECM components, Wnt and TGF-β signaling, G protein coupled receptors, cell adhesion molecules, and cytoskeletal components (reviewed in [95]). Figure created using BioRender. Citation for Figure 4: [95].

As mentioned earlier, although differing in geometry, human valve leaflets all share a similar structure in the fibrosa, spongiosa, and ventricularis layers. Changes in the cardiac physiological environment have the potential to disrupt the valve’s mechanical microenvironment at the ECM/cellular level (Figure 4). Collagen fibers mediate the load-bearing integrity of the valve. In healthy valves, collagen fibers in the fibrosa layer are primarily oriented along the circumference of the valve. Pant and colleagues (2018) investigated whether the arrangement of fibrous collagen proteins in the ECM of porcine tricuspid valve explants differed in a pressurized environment versus a non-pressurized environment. The study found that the presence of hydrostatic pressure increased the alignment of collagen fibers in the leaflets, suggesting that normal physiological pressure helps to maintain the collagen-based mechanical integrity of leaflets [89]. Conversely, abnormal pressures might contribute to disease by adversely affecting the collagen microenvironment. Since this study did not recapitulate the cyclical/oscillatory pressure waves normally present in the heart, much remains to be learned about how (or whether) differential strain on leaflets during the course of the heartbeat impacts the collagen microenvironment.

In addition to affecting alignment of macromolecules in the extracellular matrix, pressure can affect function of the VICs themselves (Figure 4). Application of pathological stretch to mature porcine aortic valve explants caused VICs to upregulate myofibroblast markers such as α-smooth muscle actin and vimentin [90]. Indeed, gene expression profiling of porcine aortic VICs cultured under conditions of high pressure demonstrated large-scale transcriptional changes compared to control cells. Of note, expression of several genes in the inflammatory response pathway was altered, as well as expression of ECM proteins [91]. Taken together, these results establish the existence of a mechanotransduction pathway between pressure and the mature valve leaflet which has the ability to substantially alter gene expression.

Due to difficulties in precisely manipulating pressure without altering other hemodynamic and physiologic variable in vivo, few studies have attempted to examine the effect of high pressure on heart valves in whole animal models. However, analysis of heart valves in giraffes provides insights. Blood pressure in the giraffe is higher than any other mammal (approximately twice that of humans) in order to sufficiently perfuse the head tissues atop the long neck. Despite these high pressures, giraffes do not develop premature valve disease. In a study to determine how giraffes have adapted to this extreme condition, Funder and colleagues noted that the aortic valve was 70% stronger and stiffer than bovine controls. Aortic leaflets were thicker and showed higher collagen and elastin content. In addition, collagen was more compactly arranged than bovine controls. Thus, although the heart mass in giraffes is comparable to other mammals (≈0.5% of body weight), giraffe hearts have adapted to their hemodynamic environment by altering the abundance and arrangement of ECM macromolecules within the valve leaflets [92].

Paracrine factors in the transforming growth factor β (TGFβ) superfamily, including BMP-2 and BMP-4, have been implicated in the pathological response of valves to elevated cyclic tissue stress. Explanted valves placed in a bioreactor to induce physiological levels of cyclic stress expressed BMP-2 and BMP-4. Under conditions of pathological stress, expression of these genes was significantly higher, particularly in the endothelial layer on the fibrosa side of the cusps. Pathological degrees of stretch induced valves to mount an enhanced osteogenic and calcification response via a BMP-dependent mechanism, but those events could be blocked by the addition of BMP antagonists to the system. These data suggest that the endothelial layer acts as the mechanotransducer of cyclic stress, and that under conditions of elevated stretch, BMP signaling is required for the process of valve calcification [93]. In another study, application of TGFβ to VICs in vitro facilitated their differentiation into myofibroblasts. The contractile valve myofibroblasts then exerted tension on the valve ECM, resulting in a dramatic realignment of fibronectin fibrils and the formation of stress fibers [94].

#### 3.2.2. Pressure as a Cue during Embryonic Valve Development

Evidence supports the link between increased pressure and valve pathology in mature organisms, but less is known about how pressure may affect the development of the valve leaflets during embryogenesis. Much of what we do know comes from surgical procedures performed on chick embryos including outflow tract (OFT) banding (sometimes called conotruncal banding) or left atrial ligation [96]. As its name implies, OFT banding involves suturing the developing OFT to reduce its cross-sectional area and consequently create a dramatic increase in afterload. On a physiological level, OFT banding produces cardiomyocyte hyperplasia in both chicken embryos and in guinea pigs. The left atrial ligation procedure redirects blood flow from the left atrium to the right atrium, thereby creating an increase in preload on the right side of the heart and a concomitant decrease in preload on the left side. This model results in an underdeveloped, hypoplastic left ventricle and a compensatory hyperplastic overdevelopment of the right ventricle [97]. Similar results are seen in humans with left outflow obstruction, with some recovery seen upon removal of the obstruction [98].

The emerging theme of these studies is that alterations in the local hemodynamic environment, producing either too much or too little pressure, significantly alter the composition of the extracellular matrix (cardiac jelly) in the endocardial cushions, and affect the propensity of endothelial cells to undergo EMT and invade the cardiac jelly. OFT banding performed on chick embryos at Hamburger–Hamilton (HH) stage 18 (approximately 3 days of incubation, during EMT) dramatically increased left ventricular pressure and blood flow velocity in the outflow region during the onset of EMT in the endocardial cushions. Along with these changes, researchers observed a significant increase in the number of cells undergoing EMT in the outflow tract region, and a substantial remodeling of the cardiac jelly near the cushion mesenchyme. Proteomic analysis demonstrated that several ECM proteins such as fibulin, mucin 6, and laminin underwent several fold changes in response to increased pressure [99].

Interestingly, outflow tract banding at later stages (Hamburger–Hamilton 21, post-EMT during ECM remodeling) resulted in smaller, dysmorphic mitral valves by Hamburger–Hamilton stage 29. Dysmorphia was accompanied by a decrease in endocardial apoptosis, and dysregulation of ECM proteins such as fibulin and collagen. Expression of shear-responsive genes such as *KLF2* and *endothelial-1* (*EDN1*) was elevated. *KLF2* was specifically upregulated on the atrial side of the mitral valve, while *EDN1* was increased throughout the right ventricular myocardium [100].

Indeed, the developmental time when outflow tract banding is performed seems to be particularly relevant to how well EMT progresses in valve-forming regions. Banding at an earlier stage (Hamburger–Hamilton 15) lead to a significant decrease in OFT cushion volume, accompanied by the production of fewer mesenchymal cushion cells. Despite higher shear rate and pressure as measured by computational fluid dynamics, expression of the shear-responsive gene *KLF2* was decreased, as measured by qPCR. Genes involved in EMT regulation, such as TGFβ, were similarly downregulated [101]. Whether the EMT defect occurred due to changes in pressure directly affecting EMT-related pathways or whether these changes are secondary to malformation of the cardiac cushions remains unclear.

The left atrial ligation (LAL) approach offers additional perspective since it produces simultaneous but distinct effects on the left and right atrioventricular valves. The right atrioventricular valve—which experiences higher pressure in embryos that have undergone LAL—becomes more fibrotic and resembles a bicuspid valve rather than its typical tricuspid morphology. The left atrioventricular valve—which experiences abnormally low pressure—was either dysplastic, or in severe cases, resembled underdeveloped atretic valves [97]. Other research noted endocardial fibroelastosis in chicken embryos that have undergone LAL, and increase in myofibroblast markers in the endocardial cushions, accompanied by an increase in collagen expression [102]. In adult valve pathology, one hallmark of a diseased valve is the differentiation of VICs to myofibroblasts, concomitant with ECM remodeling [94]. Just like Goldilocks, the developing valves need their surrounding pressure to be “just right”.

#### 3.2.3. Signaling Pathways Involved Sensing and Responding to Changes in Pressure in Valve Development

Given that afterload can affect both embryonic valve development and pathology, it is of great interest to understand the molecular mechanisms that sense and respond to changes in pressure to facilitate mechanotransduction, and whether cells use the similar genetic signals as in adult valves. As pressure in the heart is intrinsically tied to shear stress and oscillatory flow, it can be difficult to disentangle the effects of pressure from the effects of blood flow. Insight has come from in vitro studies that precisely manipulate explanted tissue to replicate changes in afterload and preload. As in adult hearts, as preload increases, the stretch of individual myocytes must increase to compensate for the increase in load. An increase in stretch can trigger a cellular response to initiate transcription of target genes. Several mechanisms have been proposed to account for how load on the heart may be transduced to endocardial and myocardial cells. Increases in afterload create increased pressure on the heart wall, which may in turn distort the glycocalyx or activate mechanosenstive proteins such as integrins, ultimately leading to differential intracellular cellular responses. The next section will describe three common pressure-sensitive pathways involved in heart valve development.

Rho/Rac cascade. Rho and Rac are membrane bound GTPase proteins that belong to the Ras superfamily. When activated by GTP binding, Rho and Rac activate downstream effects that alter the cytoskeletal properties of the cell. To date, more than 70 different effector molecules have been described for members of the Rho/Rac family. One of the best characterized is the Rho-associated serine/threonine kinase (ROCK); when activated, ROCK phosphorylates myosin light chain causing polymerization of myosin fibers. In addition, ROCK activates LIM kinase, which phosphorylates and deactivates the actin severing protein Cofilin. The combined effect of these transduction events is increased cellular stiffness and increased actomyosin contractility in the cell. Other downstream effectors of Rac include several cytoskeletal proteins including scaffolding proteins and tubulin. Additionally, Rac can stimulate a downstream kinase called p-21 activated kinase 1 (PAK1). Overall, Rho and Rac respond to mechanotransductive cues by mediating the cytoskeletal response to mechanical stress [103,104].

During valve development, Rho and Rac have opposing expression patterns in the cushion mesenchyme. In avian embryos at Hamburger–Hamilton stage 25 (post-EMT), Rho expression and activation was relatively high as determined by ELISA and immunohistochemistry, but as development progressed, expression and activation of Rho decreased. Conversely, Rac activation was relatively low at Hamburger– Hamilton stage 25, but increased through valve leaflet remodeling stages, and ultimately reached over 2-fold greater activity by Hamburger– Hamilton stage 36 [105]. Inhibition of ROCK prevented flow-driven fibrous ECM accumulation and valve stiffening in explanted HH25 avian valves, demonstrating an additional function for the mechanotransductive abilities of Rho/Rac in mediating ECM dynamics [106]. Finally, Rho-GTPases have been implicated in regulating valve interstitial cell phenotypes in adult valves. RhoA was upregulated in VIC nodules associated with calcified aortic valve disease. Inhibition of ROCK dramatically reduced expression of myofibroblast markers such as α-smooth muscle actin [107]. These observations on altered valvular cell phenotype indicate yet a third way that Rho and Rac mediate changes in response to mechanotransductive cues.

Exactly how the biomechanical environment might activate Rho and Rac remains an area of open research. One mechanism for activation of these GTPases lies in regulation of GTPase associated proteins such as Guanine Nucleotide Exchange Factors (GEFs). Often, Rho GEFs associate with other cytoskeletal elements. When those cytoskeletal elements are deformed, GEFs dissociate and activate Rho. For example, under conditions of high tension, GEF-H1 is recruited to cell adhesions, where it is activated by the Focal Adhesion Kinase (FAK) pathway. GEF-H1, in turn, activates RhoA [108]. As Rho and Rac each associate with different GEFs, they have a large capacity to associate with different cytoskeletal proteins. Thus, Rho and Rac could conceivably mediate distinct cellular responses in response to a variety of mechanical stimuli [109].

Rho and Rac have different roles in valve development, as suggested by their differential expression patterns. Overexpression of Rho is sufficient to drive AV progenitor cells to differentiate into myofibroblasts in collagen gel assays, whereas loss of Rho prevented expression of myofibroblastic genes such as α-smooth muscle actin and serum response factor. Conversely, RAC was found to be required for ECM compaction and fiber formation and overexpression of RAC promoted matrix compaction in collagen gel explants. Recently, RAC proteins were found to be activated by cyclic mechanical stretch that was dependent on a GTPase Activating Protein (GAP) called FilGAP. When placed in a bioreactor, atrioventricular valve cells cultured in collagen gels increased expression of RAC when exposed to cyclic stretch, but did not when exposed to absence of mechanical stress or constant mechanical stretch. In conditions of low mechanical stress, FilGAP was associated with the large cytoskeletal protein Filamin A. Filamin A connects transmembrane proteins to the actin cytoskeleton, and serves a variety of intracellular scaffolding functions. Under conditions of high mechanical stress, Filamin A undergoes a conformational change which releases FilGAP, which in turn activates Rac. In this way, high cyclic stretch mediates compaction and development of the valve ECM [105].

Nfat/calcineurin signaling. The Nfat/calcineurin signaling cascade is a mechanosensitive calcium-dependent signaling pathway implicated in cardiac valve development and hypertrophy. The mechanosensor in this pathway is calcineurin, which is a protein phosphatase consisting of a 60 kDA catalytic subunit (CnA) and a 19 kDA regulatory subunit (CnB). CnB has four EF-hand calcium binding sites; when is calcium is bound, calmodulin is recruited to the complex. Calmodulin recruitment causes a conformational change that exposes the catalytic phosphate site and yields a constitutively active phosphatase. The active form of calcineurin then removes a phosphate group from Nuclear Factor of Activated T-cells (NFAT), which translocates to the nucleus and affects transcriptional change [110].

Activation of calcineurin is regulated through calcium and cellular stretch. In cardiac cells, inactive calcineurin is tethered to the sarcomere Z-disc via interactions with LIM protein (MLP) and calsarcin. Under conditions of high mechanical stress, MLP dissociates from the Z-disc and translocates into the nucleus where it plays a role in initiating expression of hypertrophic genes. Knockout of MLP or pharmacological inhibition of MLP-calcienurin binding abrogated calcineurin activation in murine pressure overload models, confirming that the stretch-sensor MLP is strictly required for calcineurin signaling at the Z-disc [111,112]. In addition to its role as a mechanostransducer via scaffolding with the sarcomere, calcineurin also acts at the plasma membrane through calcium channels. Calcineurin directly binds and dephosphorylates L-type calcium channels (LTCC). Dephosphorylation increases channel activation, which results in elevated calcium levels. As calcineurin is itself regulated by calcium, this regulatory pathway likely represents a feed forward mechanism [113].

The NFAT-Calcineurin pathway is required for valve development. Tissue-specific knockout of endocardial CnB using a cre-lox mouse model resulted in failure of valve development and embryonic lethality by E14.5. Prior to lethality, mesenchymal cells were present in the valve cushions; however, valve leaflets lacking CnB displayed altered extracellular matrix and failed to elongate. Notably, myocardial NFAT-signaling at E9 is required for the initiation of EMT. Pharmacological inhibition of NFAT with cyclosporin A inhibited EMT in collagen explant gel studies. This inhibition could be rescued through addition of a Vascular Endothelial Growth Factor (VEGF) inhibitor, indicating that myocardial NFAT works to suppress VEGF signaling during endocardial cushion formation [114].

## 4. Concluding Remarks

The role of mechanotransduction in regulating valve development and pathology has become increasingly appreciated over the last few years. In several cases, the field is now able link specific fluid forces with underlying molecular circuitry that is essential for valve development progression. Several open questions remain. Firstly, much of our understanding of how hemodynamic cues operate comes from either adult organisms or from in vitro assays. This can be attributed to the difficulty of specifically manipulating only a single biomechanical cue in animal model systems, especially small embryos. The interrogation of more model systems using sophisticated tools such as optical tweezers to precisely manipulate hemodynamic forces at the developing valve could greatly enhance our understanding of how biomechanical cues affect the valve. Secondly, our understanding of the later stages of valve development remain incomplete. More work needs to be done to elucidate the mechanisms by which VICs remodel the ECM during development, and to identify the cues that cause supporting structures such as the chordea tendea to form. Thirdly, the advent of RNA-seq has been highly advantageous; we now have differential gene expression data sets from several different time points and mutants that have yielded great insight into the valve development process [33,115,116]. The next step in this process is to generate gene regulatory networks to identify the interlinking pathways that govern valvogenesis. Insight into how mechanotransduction impacts valve formation may facilitate the design of therapeutics to treat valve disease as well as improve the engineering of tissue-derived artificial valves.
